# Characterization of a mammalian prosencephalic functional plan

**DOI:** 10.3389/fnana.2014.00161

**Published:** 2015-01-06

**Authors:** Sophie Croizier, Sandrine Chometton, Dominique Fellmann, Pierre-Yves Risold

**Affiliations:** EA 3922, SFR FED 4234, UFR Sciences Médicales et Pharmaceutiques, Université de Franche-ComtéBesançon, France

**Keywords:** melanin concentrating hormone, cell cord, pioneer tracts, lateral hypothalamic area, medial forebrain bundle

## Abstract

Hypothalamic organizational concepts have greatly evolved as the primary hypothalamic pathways have been systematically investigated. In the present review, we describe how the hypothalamus arises from a molecularly heterogeneous region of the embryonic neural tube but is first differentiated as a primary neuronal cell cord (earliest mantle layer). This structure defines two axes that align onto two fundamental components: a longitudinal tractus postopticus(tpoc)/retinian component and a transverse supraoptic tract(sot)/olfactory component. We then discuss how these two axonal tracts guide the formation of all major tracts that connect the telencephalon with the hypothalamus/ventral midbrain, highlighting the existence of an early basic plan in the functional organization of the prosencephalic connectome.

## Introduction

**Figure 1:**
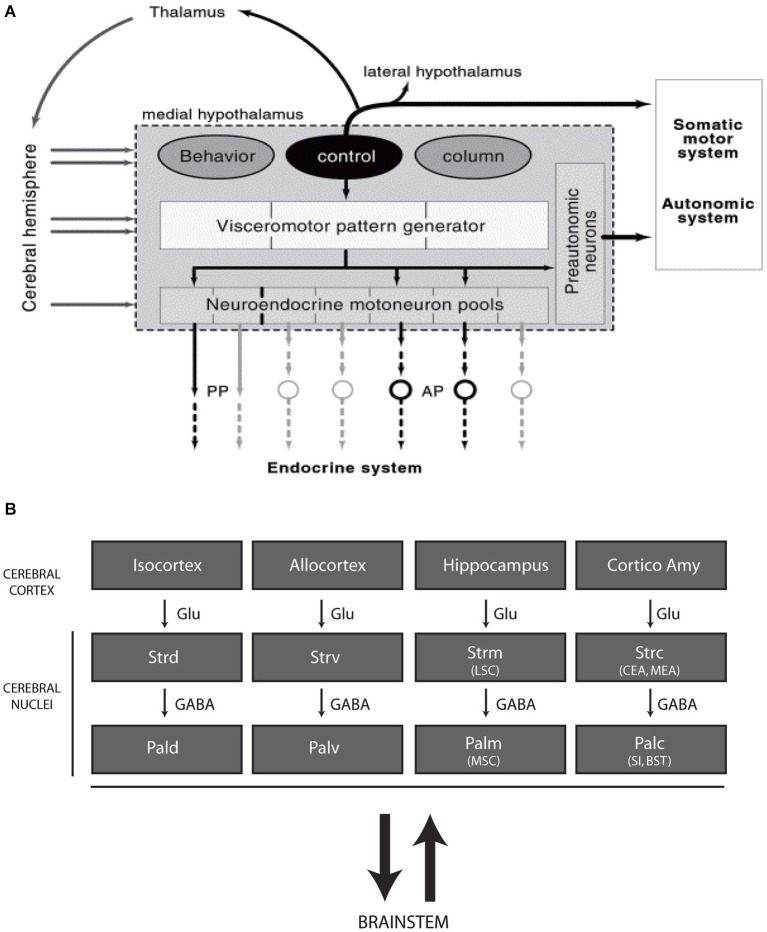
**(A)** Diagram illustrating the recent concept of the morphofunctional organization of the hypothalamus. Hypothalamic periventricular structures form a visceromotor pattern generator (VMPG) network that controls neuroendocrine and visceral responses. This VMPG is influenced by medial zone nuclei. These nuclei form a behavioral control column and are involved in the expression of goal oriented behaviors. They control somatic motor responses through projections in the tectum (periaqueductal gray), but they are also involved in closed loop circuits with the telencephalon, in particular through projections to the thalamus. Reproduced with permission from Thompson and Swanson, 2003. **(B)** Schematic representation of the organization of the telencephalon (adapted from Risold, 2004). The cerebral cortex as a whole, including the cortico-amygdaloid nuclei, topographically projects onto the striatum (dorsal, ventral, medial, and posterior divisions), which is connected with the pallidum (similarly parceled into dorsal, ventral, medial and posterior divisions). While projections from the cortex are glutamatergic, the striatum and pallidum are massively GABAergic and are bidirectionally connected with the brainstem. Abbreviations: AP: anterior pituitary; BST: bed nuclei of the stria terminalis; CEA: central nucleus amygdala; Cortico Amy: cortical nuclei amygdala; GABA: gamma aminobutyric acid; Glu: glutamate; LSC: lateral septal complex; MEA: medial nucleus amygdala; MSC: medial septal complex; Pal d-v-m-c: dorsal, ventral, medial and caudal divisions of the pallidum; PP: posterior pituitary; SI: substantia inominata; Str d-v-m-c: dorsal, ventral, medial and caudal divisions of the striatum.

As a whole, the hypothalamus is involved in an extremely large range of functions, including neuroendocrine and visceral responses, thermogenesis, circadian or seasonal cycles, sleep or general arousal, the expression of specific instinctive behaviors, the control of rhythmic cortical (hippocampal) neuron firing, emotion and reward. Therefore, the hypothalamus is a complex structure composed of dozens of cell groups or nuclei that are often involved in several of these responses. Classically, the hypothalamus has been divided into four anteroposterior regions (preoptic, anterior, tuberal and posterior regions) and three longitudinal zones (periventricular, medial and lateral zones) (Swanson, 1987). This organizational scheme has been widely accepted by anatomists and physiologists during the past decades but is not satisfactory, as most of the borders are not clear and are often arbitrarily drawn. In light of anatomical findings acquired during the late 1980s and early 1990s, the organization of the hypothalamus has been revised around the concept of a behavioral control column that is composed of the medial zone nuclei (Figure 1A; Swanson, 2000, 2005). Following this new concept, each medial zone nucleus is involved in pathways that include the tectum, thalamus and telencephalon, referencing the classical circuit described by Papez in 1937 (Papez, 1995). This new view of the hypothalamic organization is interesting as it suggests that this region is fully integrated within the complex prosencephalic networks that control behavioral expression. Therefore, the hypothalamus is capable of influencing telencephalic centers, including the cerebral cortex, as well as being influenced by descending projections (Risold and Swanson, 1996, 1997; Risold et al., 1997). Conspicuous convergences have appeared between the organization of these connections and those of the classical striato-nigral and mesotelencephalic circuits, and a revision of the telencephalic organization has been proposed (Figure 1B; Risold et al., 1997; Swanson, 2000, 2003; Risold, 2004).

Although interesting, this concept primarily involves the medial zone nuclei at hypothalamic levels but ignores large sections of this structure, especially the entire hypothalamic lateral zone (lateral hypothalamic area, LHA) (Figure 1A). The LHA is a poorly differentiated region that has always been viewed as a rostral extension of the brainstem reticular formation or a bed nucleus of the medial forebrain bundle (mfb). The mfb is the major fiber tract of the basal prosencephalon that passes through the LHA and bidirectionally connects more than fifty cell groups in the brainstem and telencephalon (Nieuwenhuys et al., 1982; Swanson, 1987). The mfb is a specific attribute of the LHA throughout its entire alar-basal extent. However, the LHA is not homogeneous. The basal portion of the LHA contains abundant cell populations that are characterized by the expression of specific peptides, such as melanin-concentrating hormone (MCH) and hypocretins/orexins, and widespread projections from the cerebral cortex to the spinal cord.

Tremendous progress in expanding the general knowledge of the forebrain embryonic development has been made over the last twenty years. Recent studies, alongside anatomical data, have led to a better understanding of the organization of the vertebrate forebrain, which has allowed for a better comprehension of its evolution (Puelles, 2001; Aboitiz, 2011). To understand the organization of the basal portion of the LHA, our group analyzed the comparative anatomy and development of hypothalamic neurons that produce MCH (Croizier et al., 2013). In the present analysis, we revised some of these observations from past and recent developmental studies regarding the forebrain to better understand the relative role of the LHA within the context of a putative general prosencephalic framework. We observed that the whole ventral prosencephalon is organized around a precocious structure, previously named the cell cord and from which the LHA differentiates, in a timely manner. This primary structure defines two axes that align onto two fundamental components: a tractus postopticus (tpoc)/retinian component and a supraoptic tract (sot)/olfactory component. These two axes determine the path of the mfb and provide what can be described as a basic structural framework for a prosencephalic “functional plan”.

## Developmental gene patterns and hypothalamic subdivisions

**Figure 2:**
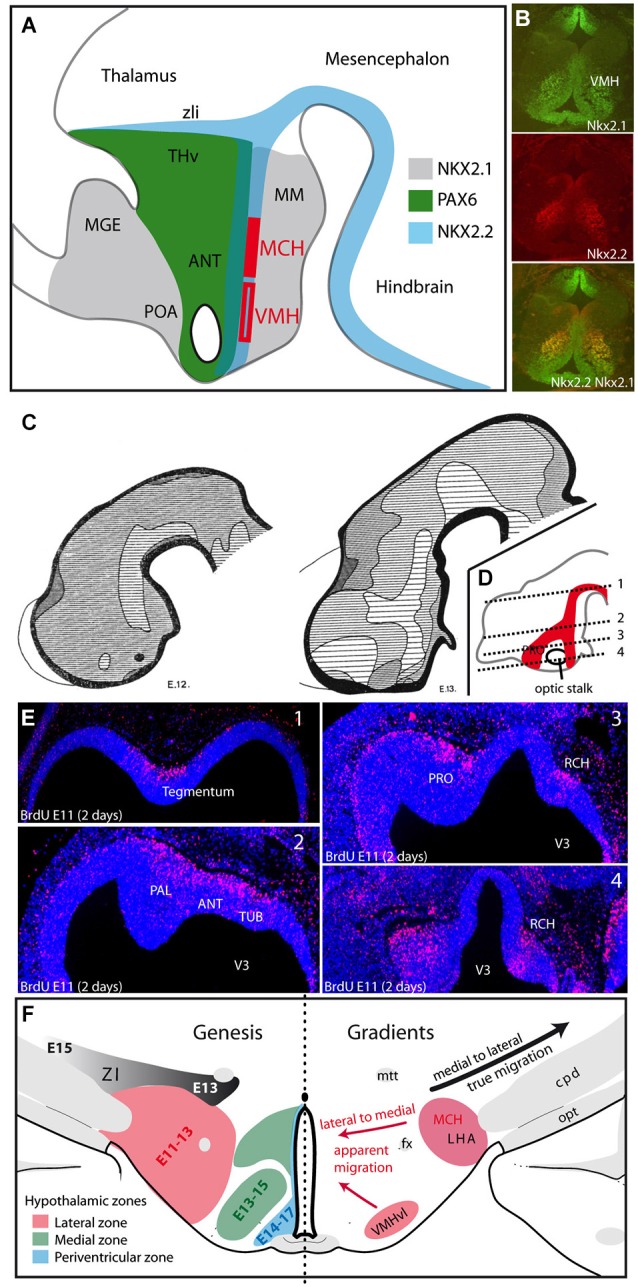
**(A)** The distribution patterns of transcription factors agree well with the alar-basal divisions of the hypothalamus into preoptic, anterior and posterior/postoptic regions. However, these patterns extend outside the borders of the hypothalamus, involving the ventral telencephalon, prethalamus-ventral thalamus and ventral midbrain. MCH neurons and neurons of the VMH are generated from Nkx2.1/Nkx2.2 expressing neuroepithelial zones in the postoptic region. **(B)** Based on immunohistochemical analysis of a horizontal section of an E15 rat embryonic hypothalamus, the VMH clearly express both Nkx2.1 and Nkx2.2. **(C)** Figures from Keyser (1972) illustrating the differentiation of the early neurogenic zone in E12 and E13 Chinese hamster embryos: the first appearance of a longitudinal zone in the ventral mesencephalon and dorsal hypothalamus correspond to the cell cord **(A)**. At E13, neurogenesis involved larger regions in the preoptic/ventral telencephalon; in other figure from Keyser that is not shown here, this author observed these regions forming one single continuum (as in **D**). This continuum takes the shape of an inverted Y. **(D,E)** Figure adapted from Croizier et al., 2011 illustrating the distribution of neurons generated at E11 on an E13 rat embryo. BrdU was injected into the pregnant dam at E11, and embryos were taken 2 days later at E13. BrdU was detected by immunohistochemistry on horizontal sections. The distribution pattern of these nuclei is schematized on a sagittal section in **(D)**. BrdU-labeled nuclei follow an inverted Y pattern. In **(E)** pictures are arranged from dorsal (1) to ventral (4). **(F)** Gradients of neurogenesis in the ventral diencephalon. Left side: Schematic representation of the neurogenic gradients in the hypothalamus and prethalamus-ventral thalamus, as described by Altman and Bayer (1986). The LHA is generated between E11and E13, the medial hypothalamus from E13 to E15 and the periventricular zone from E14 to E17. Note the medial to lateral gradient in the prethalamus-ventral thalamus (generated from E13 to E15). Right side: Drawing summarizing the gradients in the ventral diencephalon: lateral to medial gradients (red arrows) in the hypothalamus suggest the apparent or passive migrations of neurons in lateral territories (LHA or VMHvl), but the prethalamus-ventral thalamus requires the effective migration of cells away from the ventricular surface (black arrow). Abbreviations: ANT: anterior hypothalamic area; cpd: cerebral peduncle; fx: fornix; LHA: lateral hypothalamic area; MCH: melano-concentrating hormone expressing neurons; MGE: medial ganglionic eminence; MM: mammillary body; mtt: mammillothalamic tract; opt: optic tract; PAL: pallidum; POA: preoptic area; PRO: presumptive preoptic area; RCH: retrochiasmatic region; THv: prethalamus or ventral thalamus; TUB: presumptive tuberal hypothalamic region; VMH: ventromedial hypothalamic nucleus; VMHvl: ventrolateral part of the VMH; ZI: zona incerta; zli: zona limitans intrathalamica; V3: third ventricle.

**Figure 3:**
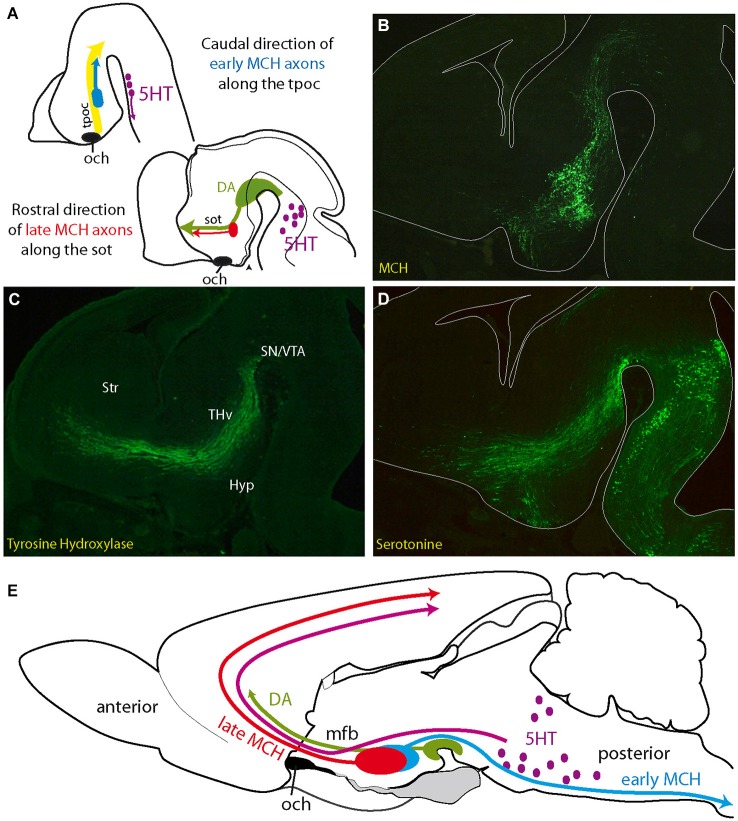
**(A)** Initial projections of MCH expressing neurons follow pioneer tracts, but their direction changes as the embryo matures. Axons of first generated MCH expressing neurons follow the tpoc toward the midbrain. Axons from late generated MCH expressing neurons follow the sot toward the telencephalon, along DA and 5HT axons (see Croizier et al., 2011). **(B–D)** Distribution of serotonin, MCH (MCH-GFP, revealed with an anti-GFP antibody; see Croizier et al., 2011) and tyrosine hydroxylase (dopamine) in three adjacent sections cut in the parasagittal plane and passing through the mfb of an E14 mouse embryonic brain. Serotonergic and dopaminergic axons from respectively the hindbrain and midbrain travel along the tpoc and arch rostrally at the level of the posterior hypothalamus, where MCH expressing cells are found. Note the pattern of serotonergic axons that closely follows the inverted Y pattern of the sot/tpoc. **(E)** Schematic illustration of the ascending serotonergic and dopaminergic pathways to the telencephalon through the mfb and the distribution patterns of early and late MCH projections in the adult rat central nervous system. Abbreviations: DA: dopaminergic neurons; Hyp: hypothalamus; MCH: melano-concentrating hormone containing neurons; mfb: medial forebrain bundle; och: optic chiasm (or presumptive position of the optic tract in **A**); SN: substantia nigra; sot: supraoptic tract; Str: presumptive striatal region; THv: ventral thalamic region; tpoc: tractus postopticus; VTA: ventral tegmental area (presumptive); 5HT: serotonergic neurons.

Very complex molecular interactions occur at the origin of the hypothalamic regions, which are very heterogeneous, even at the earliest stages. These patterns have been extensively analyzed in many more detailed works to which the reader may refer (Shimamura et al., 1995; Nieuwenhuys et al., 1998; Puelles et al., 2012). Longitudinal and transverse axes in the embryos have been revised on the basis of these patterns of gene expression (Puelles et al., 2012). Although the terminology proposed by Puelles and Rubenstein is widely used in the developmental field (see for instance “prethalamus”), in the field of adult neuroanatomy the traditional axes and nomenclature are maintained. Since we strive to be clear for all interested readers, “adult” or “developmental”, we often hesitate to use one or the other name for a structure or spatial relation. For this practical reason, and in particular to respect the main information flows in the adult brain, we have used sometimes similar rostrocaudal and dorsoventral axes in the embryonic brain as are used in the adult brain (for example see in Figure 3). We also use the terminology prethalamus-ventral thalamus, in this way adding “prethalamus”, as is often done in developmental studies, for the presumptive regions of the zona incerta and the ventral lateral geniculate nuclei. The preoptic region (POA) (actually a part of the telencephalon, see below), the anterior region (or alar hypothalamus) that is supraoptic and regions that are posterior (basal) or postoptic represent no problem (Figure 2).

From the general literature in this field one important observation retained our attention: very early in development, these gene expression patterns bear resemblances to and are contiguous with those in structures adjacent to the hypothalamus, which suggests that some hypothalamic boundaries are not sharply delineated (Figure 2A). Consider the following examples.

### The hypothalamic/telencephalic border

The POA shares many characteristics and developmental expression patterns with the pallidum, and has been considered a telencephalic structure (Moreno and González, 2011; Puelles et al., 2012). The preoptic anlage expresses the telencephalic marker Foxg1, distinguishing it from hypothalamic structures. Shh and Nkx2.1 are co-distributed in the POA, but their expression patterns extend into the pallidal anlage. Although the POA expresses Shh, it does not express Nkx2.2, whereas the hypothalamic regions do express Nkx2.2. Defining the telencephalo-hypothalamic limit has always been problematic, despite functional and connectional studies in adult animals that have been interpreted in favor of including the POA as part of the hypothalamus (Swanson, 1987; Risold et al., 1997). Earlier authors often viewed this region to be a medial and unevaginated section of the telencephalon or telencephalon impar (His, 1893; Herrick, 1910; Kuhlenbeck, 1929). These early claims have been strengthened by recent studies of gene expression patterns (Moreno and González, 2011). This definition is further reinforced by recent findings that, similar to the lateral and medial ganglionic eminences (LGE and MGE, respectively), the POA produces GABAergic (gamma aminobutyric acid) interneurons that tangentially migrate into the cerebral cortex (Brown et al., 2011; Gelman et al., 2011; Vitalis and Rossier, 2011). In addition, migrating cells from the olfactory epithelium colonize the medial septal and preoptic areas. Many of these cells express GnRH (gonadotropin-releasing hormone) and are neuroendocrine neurons, including in the septal region. Neuroendocrine functions are a hallmark of the hypothalamus, but a putative neuroendocrine zone extends beyond the actual rostral border of the hypothalamus. *Therefore, a putative hypothalamo/telencephalic limit at the septal/preoptic border is not delineated by the distribution pattern of neuroendocrine GnRH neurons, the origin of GABAergic interneurons migrating into the pallium, or developmental gene expression patterns*.

### Anterior hypothalamus/prethalamus-ventral thalamus

At early embryonic stages, Pax6 is expressed in a continuum that includes the presumptive prethalamus-ventral thalamus and extends into the optic vesicle (Stoykova et al., 1996; Puelles et al., 2013). In the hypothalamus, this pattern involves a strip of tissue between the optic stalk and the prethalamus-ventral thalamus (Figure 2A). The *Pax6* expression pattern (encompassing the alar hypothalamus and prethalamic eminence) clearly suggests an elongation of the primary embryonic brain (see the interesting paper of (Suzuki et al., 2014) about the rise of the eyes in chordates). As previously noted, Pax family members are involved in the formation of the retina and are also involved in the guidance of retinal projections and the differentiation of retinorecipient structures into the suprachiasmatic nucleus (SCN). In vertebrates, such as lampreys or batrachians, the SCN is adjacent to the prethalamus. In the anuran, Dominguez confirmed this close and continuous positioning of the anterior hypothalamus and prethalamus (Dominguez et al., 2013). In some species, projections from the prethalamus and from the tectum reach the retina through the optic tract. In mammals, the ventral geniculate body has retained a strong retinal afferent, and the intergeniculate leaflet, a small structure of prethalamic-ventral thalamic origin, has strong bidirectional connections with the SCN, reminiscent of the adjacent positions of the SCN and the prethalamus in non-amniote vertebrates (see the recent work of Suzuki et al., 2014 as well). *Therefore*, *early Pax6 expression patterns prefigure optic related pathways that in the alar hypothalamus and in the prethalamus-ventral thalamus*.

### The hypothalamus as a rostral structure

Also of particular interest for the development of the hypothalamus are the longitudinal expression patterns of Shh and Nkx2.2, which label a band of hypothalamic neuroepithelial tissue that rostrally extends from a similar band in the ventral mesencephalon (Shimamura et al., 1995; Alvarez-Bolado et al., 2012). When the anterior neuropore closure occurs, the initial expression patterns of Shh and Nkx2.2 involve the differentiating zona limitans intrathalamica (zli), at the junction between the prethalamus and thalamus. At roughly the same stage, corresponding to the beginning of neurogenesis, Shh expression (but not Nkx2.2) appears in a telencephalic region. The domain of Nkx2.2 overlaps partially with both Pax6 and Nkx2.1 expression domains (Croizier et al., 2011). The Pax6/Nkx2.2-rich region gives rise to anterior hypothalamic structures whose composition in the adult are not yet completely clear. However, the Nkx2.1/Nkx2.2 regions give rise to vast portions of the basal hypothalamus. We have clearly shown (Croizier et al., 2011) that neurons that produce MCH are generated and differentiate under the control of Shh (Szabó et al., 2009; Alvarez-Bolado et al., 2012) in this sector of the embryonic wall. In the model of Puelles, this is the RTu-I portion of the basal hypothalamus (Puelles et al., 2012); in this way, the MCH cells would represent a precocious peduncular superficial derivative of the dorsal retrotuberal basal domain.

The ventromedial hypothalamic nucleus is produced by a more rostral portion of the Nkx2.1/Nkx2.2 region (Figure 2B, and see (Altman and Bayer, 1986) for the origin of this nucleus). Shimogori identified this region as the “intrahypothalamic diagonal”, on the basis of multiple gene expression patterns (“diagonal”, that is, neither columnar nor prosomeric, but somewhat in the middle of both, rather confusingly) (Shimogori et al., 2010). The Nkx2.2 and Shh expression patterns extend into the brainstem and are known to be involved in the genesis of other very early defined neurons, including serotonergic neurons, which have diffuse projection patterns similar to MCH neurons (Ye et al., 1998). Somewhat later, Shh is also involved in the differentiation of dopaminergic ventral midbrain neurons (Riddle and Pollock, 2003; Perez-Balaguer et al., 2009). *Therefore, the co-expression of two primary markers of the basal neural tube extends into the postoptic (i.e., basal) hypothalamus*, and we observe the early production of specific neuron populations with diffuse projection patterns as MCH and serotonergic neurons in corresponding hypothalamic regions and hindbrain.

The mammillary nuclei and regions of the very ventromedial hypothalamus (VMH, arcuate nucleus) are generated by an Nkx2.1 expressing neuroepithelial zone (Puelles and Rubenstein, 2003). This appears to be the only pattern that does not show any sign of extension outside of the hypothalamic borders (although, see Puelles et al., 2013).

From all these observations we can conclude that the hypothalamus has diverse origins. Patterns of gene expressions are very complex, and precise combinations of gene expression are associated with specific cell groups or nuclei. However, at the very early stages, the patterns of Shh, Pax6, Nkx2.2 and Nkx2.1 expression indicate that the POA is a part of the telencephalon (Puelles et al., 2012) and the anterior (alar hypothalamus) and postoptic regions (basal hypothalamus) share some gene expression patterns with the prethalamus and the ventral (basal) brainstem.

## Early neurogenesis in the hypothalamus—evidence of a primary structure

In the embryonic neural tube, neurogenesis (neuron production and therefore the formation of a postmitotic “mantle layer”) begins in the ventral hindbrain, behind the cephalic flexure. This neurogenic zone extends both caudally and rostrally. In more rostral regions, Keyser very precisely depicted the patterns of morphologic modifications that occur in the hypothalamic periventricular and mantle layers of the Chinese hamster (Keyser, 1972), describing the development of a “matrix” that can be translated into a very dynamic view of the pattern of neurogenesis in the diencephalon (Figure 2C, right and left diagrams). These observations by Keyser can easily be correlated with neurogenesis studies using tritiated nucleotides or BrdU. Keyser showed that the early pattern of neuron production is not uniform throughout the hypothalamus. Neurogenesis begins in a column of cells that was named the “cell cord” by Gilbert in 1935 in the human embryo (cited in Keyser, 1972), and it was more recently observed again in the mouse embryo by Croizier (Croizier et al., 2011; Figures 2D,E). The position of the cell cord on the model of Puelles is probably basal, immediately under the alar-basal boundary (Puelles et al., 2012). This column of early neurogenesis gives rise to the first generated hypothalamic neurons that ultimately form the postchiasmatic lateral hypothalamus, and extends into the ventral midbrain. MCH expressing neurons are among the first generated cells in this region, and we showed their early differentiation within this cell cord (Croizier et al., 2011). From this original sector, neurogenesis involves more rostral territories (presumptive entopeduncular nucleus (Altman and Bayer, 1986)). Therefore, the early mantle layer forms the shape of an inverted Y in the hypothalamic primordium (Figures 2C,D). The vertical (postoptic) limb and stem of the Y are longitudinal and correspond with the Shh/Nkx2.2/Pax6 expression patterns. The supraoptic arm, however, does not respect these longitudinal patterns and is transversally oriented (longitudinal and transverse here in the sense of the model by Puelles et al., 2012). A chronological correlation could be made between the development of this supraoptic arm and the differentiation of the zli (another transverse feature), the differentiation of the telencephalic vesicle or the telencephalic expression of Shh. However, causal links have not yet been demonstrated.

Keyser also observed that the development of the mantle layer extends from these hypothalamic initial regions, in both the ventral (hypothalamic) and dorsal (ventral thalamic/prethalamic) directions (Keyser, 1972). Altman and Bayer described a lateral to medial gradient of neurogenesis in the hypothalamus (lateral to periventricular) that is clearly in agreement with the observations of Keyser (Figure 2F; Altman and Bayer, 1986). A similar gradient was also clearly demonstrated for MCH expressing neurons (Brischoux et al., 2001; Croizier et al., 2010). On the contrary, both Altman and Bayer (1986) in the rat, and Keyser (1972) in the Chinese hamster, described a medial to lateral gradient of neurogenesis in the prethalamus that generates the zona incerta (adult ventral thalamus). This gradient is opposite to the hypothalamic gradient, although both structures are generated during the same period (between E11 and E16 in the rat). Therefore, these two opposing gradients involving two adjacent structures, lateral to medial for the hypothalamus and medial to lateral for the zona incerta, clearly designate a unique sector of origin, and the region giving rise to the cell cord is a good candidate. The opposite (lateral-medial and medial-lateral) gradients of genesis in the hypothalamus and prethalamus-ventral thalamus suggest distinct strategies of cell migration; the lateral to medial hypothalamic gradient suggests a dominant passive migration, as was shown for MCH expressing neurons in the dorsal hypothalamus and is also evident for the VMH. However, the medial to lateral gradient of the zona incerta indicates that lateral neurons must actively migrate far from the ventricular surface (Figure 2F; Keyser, 1972; Altman and Bayer, 1986).

Therefore, the initial neurogenesis in the hypothalamus produces a primary inverted Y-shaped structure named here the cell cord (although this structure is slightly different but encompasses the original cell cord). The prethalamus-ventral thalamus and medial hypothalamus are then produced through inverted gradients that are dorsal and ventromedial to this initial cell cord, respectively.

## The structural wiring of the hypothalamus and the chronotopic differentiation of the whole prosencephalon

Gene expression patterns indicate that the development of the hypothalamus is a multifactorial process, but gradients of neurogenesis show that time is a key parameter. The study by Altman and Bayer (1986) emphasized this point when these authors described three waves of genesis to form the three longitudinal zones of the hypothalamus, even if “three waves” might not to be literally considered (Alvarez-Bolado et al., 2012). Time is also a critical parameter according the description of the “matrix” (mantle layer) and cell cord by Keyser (1972). Relying on the development of MCH expressing neurons, we have more recently illustrated the importance of chronology in the organization of this conspicuous neuron population in the posterior hypothalamus in both rat and mouse (Croizier et al., 2010, 2011). Therefore, an analysis of the hypothalamic wiring is inseparable from the timely and sequential events that lead to the morphofunctional organization of the whole prosencephalon.

Tract formation in the hypothalamus immediately follows neurogenesis and accompanies the differentiation of hypothalamic regions. The main axonal bundles of the prosencephalon, transverse and longitudinal, have been illustrated on the prosomeric model by Puelles et al. (see their Figure 8.34) (Puelles et al., 2012). Pioneer tracts have been well described in a series of papers (Herrick, 1910; Easter et al., 1993; Mastick and Easter, 1996). Pioneer tract organization is very well conserved in the young embryo of all vertebrates, from fishes to mammals. The tpoc is the first prosencephalic tract. It is composed of commissural axons (postoptic commissure) and axons running toward the ventral midbrain, parallel to the Nkx2.2 expression domain. This tract joins the medial longitudinal fasciculus (mlf) in the midbrain. The sot and the stria medullaris are formed in the preoptic/entopeduncular primordium. The sot joins the tpoc by passing over the optic stalk (Anderson and Key, 1996), while the stria medullaris runs toward the dorsal diencephalon. The long projections of the hypothalamus subsequently organize along these pioneer tracts, and several stages can be recognized. *Each stage is correlated with a different degree of organization in the embryonic brain and is reflected in the structure of the adult hypothalamus*.

– The first stage is concerned with the initial formation of the pioneer tracts. Guidance cues, such as Slit/ROBO family members, and transcription factors, such as Pax6, play important roles in constraining the paths of pioneer tracts (Mastick et al., 1997; Nural and Mastick, 2004; Ricaño-Cornejo et al., 2011). Both tpoc and sot clearly recall the early neurogenic pattern and travel along the inverted Y-shaped cell cord.
  Initially, these tracts are composed of descending axons. The first MCH expressing neurons and the first neurons in the ventrolateral VMH are generated during this preliminary stage (Figure 3A). MCH and SF-1 are expressed in neurons within the dorsal and ventral cell cords, respectively, and their axons have been traced in the tpoc running toward the mesencephalon (Croizier et al., 2011; Cheung et al., 2013). In the adult hypothalamus, this first stage is represented by spinally projecting MCH neurons (Brischoux et al., 2001; Croizier et al., 2010, 2011) that are located very laterally in the rat LHA (Figure 3E). Neurons in the lateral region of the adult VMH, where the first SF1-labeled cells settle, send abundant projections through the supraoptic commissures (Canteras et al., 1994), and this pattern is also clearly reminiscent of descending SF-1 projections in the tpoc.
– The second stage is characterized by the growth of ascending projections along the tpoc and sot. This growth is particularly well illustrated by the differentiation of ascending projecting MCH expressing neurons (Figure 3A). During this stage, large bundles of ascending axons containing neurotransmitters, such as serotonin and dopamine, develop from the brainstem. The projections from hindbrain serotonergic or ventral midbrain dopaminergic neurons are initially longitudinal as they follow the tpoc, changing course in the basal hypothalamus and becoming transversally oriented in order to migrate towards the telencephalon (Figures 3B–D). MCH expressing neurons settle in the hypothalamic region, where these axons change direction. Moreover, in the rat, the phenotype of MCH expressing neurons change drastically as the mesotelencephalic dopaminergic pathway develops. As mentioned above, the first MCH expressing neurons send descending axons to the spinal cord. However, MCH expressing neurons produced during the second stage, as the dopaminergic mesotelencephalic axons progress in the mfb, project axons toward the telencephalon but not the spinal cord in the adult rat (Brischoux et al., 2002; Croizier et al., 2010, 2011; Figure 3E).
  The mechanisms responsible for the change in the axial organization of the MCH population appear to be related to the differentiation of the telencephalic vesicles (Croizier et al., 2011). The growth and differentiation of telencephalic vesicles involves complex interactions between morphogenic molecular actors, such as Fgf8 and Wnts (Rubenstein et al., 1998; Aboitiz, 2011). These proteins are produced by organizing centers and may diffuse and act far from their production sites. Croizier also detected a sharp increase in the production of the chemoattractant Netrin1 in the telencephalon following the onset of neurogenesis (Croizier et al., 2011). Therefore, the telencephalon exerts a strong influence on the developing rostral brainstem as it differentiates. This influence likely increases as the rostral brainstem becomes involved in very active neurogenesis (Figure 4A). Although not fully understood, the telencephalic organizing centers, such as the ventral and cortical hems, and the diencephalic organizing centers, such as the zli, interact through the production of morphogenic protein gradients (Marín et al., 2002; Pottin et al., 2011; Rash and Grove, 2011). These processes are important for the coordinated growth of the cortex and thalamus and for the establishment of corticothalamic and thalamocortical connections. However, the second stage also corresponds to an outburst of neurogenesis throughout the hypothalamus and the ventral mesencephalon. The dopaminergic neurons of the substantia nigra and ventral tegmental area are representative of this second stage. Their soma migrate through pre-existing premotor or motor midbrain structures, such as the Edinger-Westfall or oculomotor nuclei, to settle in the ventral midbrain. Their ascending projections through the lateral mfb to the striatum define them anatomically. The outburst of neurogenesis in the hypothalamus includes cortically projecting MCH expressing neurons and most of the hypothalamic medial zone that is generated between E13 and E15 in the rat (Altman and Bayer, 1986). During the same stage, Cheung reported ascending SF-1 expressing axons from the VMH in the mfb (Cheung et al., 2013).
  **Figure 4:**
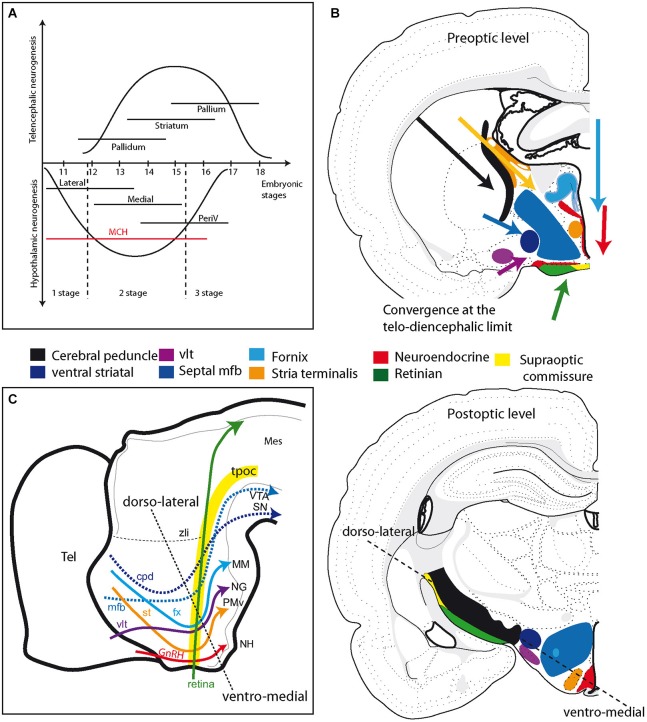
**(A)** Schematic comparison of neurogenesis in the hypothalamus and telencephalon in the rat (see text for details). Neurogenesis in the hypothalamus is described as involving three stages: an early stage that produces only the lateral zone; a second that is concomitant to neurogenesis in the telencephalon and produces neurons in all hypothalamic longitudinal zones, but mostly the medial; a late third stage that concerns mainly periventricular zone neurons. Note that MCH neurons are produced during all three stages. **(B)** Illustration of the primary fiber tracts in the hypothalamus; these tracts originate in the telencephalon or retina and converge at the diencephalon-telencephalon limit at preoptic level (top drawing). In the postoptic hypothalamus (bottom drawing), these tracts are all aligned according to an axis determined by the dashed line. See text and supplementary information for details. **(C)** Schematic representation of the primary descending tracts in the ventral prosencephalon on a sagittal view of the embryonic brain. The tracts are topographically organized. Descending pathways from the telencephalon end in more posterior regions as they are distributed more dorsally in the hypothalamus. The dashed line recalls the ventro-medial/dorso-lateral axis, as in **(B)**. Abbreviations: cpd: cerebral peduncle; fx: fornix; GnRH: gonadotropin-releasing hormone; MCH: melano-concentrating hormone; mes: mesencephalon; mfb: medial forebrain bundle; MM: mammillary body; NG: nucleus Gemini; NH: neurohypophysis; periV: periventricular; PMv: ventral premmamillary nucleus; SN: substantia nigra; st: stria terminalis; tel: telencephalon; tpoc: tractus postopticus; vlt: ventrolateral hypothalamic tract; VTA: ventral tegmental area; zli: zona limitans intrathalamica.
  Therefore, the second stage in the development of the hypothalamus is characterized by the differentiation of most of the hypothalamic lateral and medial cell groups and is concomitant with the differentiation of the dopaminergic ventral midbrain.
– The third stage corresponds with the differentiation of the thin periventricular hypothalamic zone and neuroendocrine pathways, as neurogenesis reaches exhaustion in the hypothalamic anlage. Most of the late produced periventricular structures are regions of the visceromotor pattern generator (VMPG) that was described by Thompson and Swanson (Thompson and Swanson, 2003). These structures are often perichiasmatic or involve dorsal hypothalamic cell groups (dorsomedial nucleus, dorsomedial capsule of the VMH). The last generated MCH expressing neurons in the rat are periventricular and project to the arcuate nucleus (Croizier et al., 2010), and some others can be characterized as neuroendocrine in nature (Cvetkovic et al., 2003). These last produced MCH expressing neurons can be viewed as part of the VMPG. Therefore, most of the VMPG and LHA could have a common origin in the periventricular dorsal hypothalamus but be generated at different periods.
– Finally, perinatal processes that are not further evocated here and that are associated with the functional organization of the hypothalamus might constitute a fourth stage. For example, the morphological modifications induced by hormones, such as sexual steroids or leptin, are now well described during the perinatal period (Bouret et al., 2004; Simerly, 2005; Ciofi et al., 2007).

## Hypothalamic tract topographic organization

The anatomical dispositions of all the major tracts crossing or bounding the hypothalamus of the adult animal are summarized in Figure 4B and supplementary information. From this, it appears that all of the major tracts that originate in the dorsal or ventral telencephalon or in the retina converge in the anterior hypothalamic region (alar hypothalamus). However, upon reaching the caudal hypothalamus, they all follow adjacent pathways. This topographical organization closely follows the initial scaffolding provided by the tpoc in the caudal hypothalamus, as well as that provided by the sot for descending tracts from the telencephalon. The more ventral of these tracts (fornix, stria terminalis, ventral lateral hypothalamic tract, neuroendocrine tracts) end in the neurohypophysis and caudal hypothalamus, while the others (mfb, cerebral peduncle) take divergent routes at the mesencephalic limit. The relative path of these tracts can be illustrated on a schematic sagittal view of the embryonic brain (Figure 4C). We therefore observed that the descending tracts from the telencephalon traveled along a transverse path related to the sot, but as they joined the optic tract at the level of the posterior hypothalamus, their course became longitudinal, coincident with that of the tpoc and optic tract. Only the stria medullaris escaped from this general scheme.

## Structural organization of the hypothalamus and the prosencephalic functional plan

**Figure 5:**
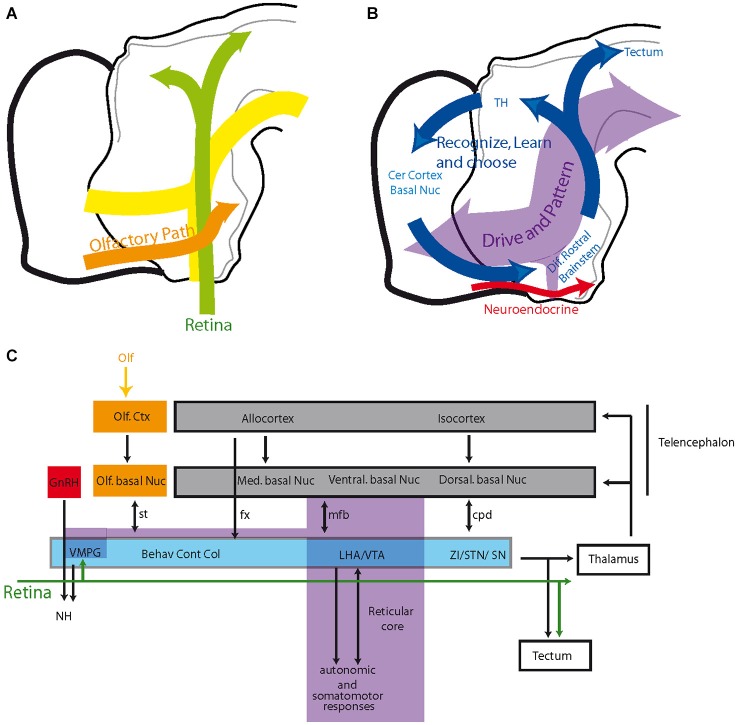
**(A)** Schematic organization of olfactory and optic pathways in the hypothalamus compared to the cell cord. **(B,C)** Organization of the prosencephalic connectivity. The hypothalamus and ventral midbrain are engaged in circuit loops (blue in **B**) that involve topographically organized descending inputs from the telencephalon and topographically organized outputs to the thalamus and tectum. These circuits play roles in behavioral expressions, voluntary motor responses and learning/memory. They are superimposed on a reticular core (including the VMPG), which is rostrally contiguous to the reticular brainstem, recalling the scaffold of the original cell cord, and are mostly involved with driving and patterning brain activities. See text for details. Abbreviations: Behav Cont Col: behavioral control column (medial zone nuclei of the hypothalamus); cer: cerebral; cpd: cerebral peduncle; ctx: cortex; fx: fornix; GnRH: gonadotropin-releasing hormone; LHA: lateral hypothalamic area; med: medial; mfb: medial forebrain bundle; NH: neurohypophysis; nuc: nucleus; olf: olfactory; SN: substantia nigra; st: stria terminalis; STN: subthalamic nucleus; TH: thalamus; VMPG: visceromotor pattern generator; VTA: ventral tegmental area; ZI: zona incerta.

The development of the hypothalamus and the adjacent “prethalamus-ventral thalamus” involves several stages. The first stage includes the differentiation of the cell cord. The inverted Y-shaped arrangement is the first differentiated structure of the prosencephalon and guides the first pioneer tracts, including the tpoc and sot. Following the differentiation of the cell cord, neurogenesis becomes generalized in the prosencephalon, preceding the formation of all of the major fiber tracts that connect the telencephalon with the hypothalamus and mesencephalon. Therefore, as new neurons settle medially or dorsally to the cell cord, fiber tracts topographically organize dorsally or medially to the early mfb. The tpoc also guides the optic tract and the supraoptic commissures. Therefore, while the supraoptic arm of the initial inverted Y-shaped pattern appears to guide the olfactory projections in the hypothalamus, the postoptic guides tracts parallel to retinal projections (Figure 5A).

These first and second stages in the forebrain differentiation leave traces that are found in the adult anatomical organization of the hypothalamus. In the adult brain, neurons that were initially derived from the cell cord form a large part of the reticularly organized hypothalamus, including the LHA. This structure can be considered, at a functional level, a rostral extension of the primary medial mantle layer that originates in the hindbrain and from which serotonergic neurons are produced. The concept of a deep structure in the brain with a reticular like appearance that is involved in general arousal in all vertebrates and that forms a reticular core is quite ancient. For example, this reticular core was termed the isodentritic core by Ramon-Moliner and Nauta and was also compared to the deep ancient brain described by McLean (Ramón-Moliner and Nauta, 1966; Nieuwenhuys et al., 1998; Swanson, 2003). Pfaff recently argued that primitive mechanisms involving the reticular formation of all vertebrates are important for initiating the activation of behaviors (Pfaff et al., 2012). The hypothalamic cell cord along with a more caudal cell cord are reminiscent of such primitive structures. In the adult brain, structures along the cell cord serve synchronizing and patterning functions; MCH and the cognate hypocretin expressing neurons play roles in the sleep/wake cycle. MCH knockout mice have modified locomotor activities, and in humans, the absence of hypocretin in the dorsal hypothalamus is associated with narcolepsy (Peyron et al., 2000; Verret et al., 2003). This cell cord could also have important functions during all stages of brain development. Serotonergic neurons, which are among the very first generated cells in the hindbrain, act as pacemakers to synchronize the electrical activity of local motoneurons (Moruzzi et al., 2009). Later, the sot and tpoc are the precocious frames for the mfb. The early mfb contains dopamine and serotonin projections, and both neurotransmitters play key roles in the development of telencephalic structures. Dopamine modulates the cell cycle and proliferation in the ganglionic eminences and influences the maturation of the local circuitry (Diaz et al., 1997; Goffin et al., 2010). Serotonin has well recognized developmental effects. Alterations of early serotonergic or dopaminergic pathways lead to pathological conditions, such as autism or schizophrenia (Herlenius and Lagercrantz, 2001; Kinast et al., 2013). MCH is also suspected to have trophic actions (Cotta-Grand et al., 2009).

However, the second stage of forebrain development is more specifically associated with the differentiation of the hypothalamic medial regions and structures belonging to the classical striato-nigral pathways. These structures share genetic, topographic and chronotopic characteristics during development. This developmental stage coincides with the concept of a convergence in the anatomical organization of circuits involving these regions in the adult. It therefore becomes very attractive to describe the circuits connecting the pallium, striatum, pallidum and rostral brainstem as a series of parallel interacting loops (Figures 5B,C). Alexander described several putative circuits involving dorsal and ventral striatal components (Alexander et al., 1990). The Swanson group described several other circuits, involving medial and posterior striatal/pallidal structures and hypothalamic medial zone nuclei, suggesting the existence of a basic organizational plan (see Section Introduction; Swanson, 2003; Thompson and Swanson, 2010). We believe that we can now hypothesize that these sets of circuits are developmentally linked and can identify this as to be basic mammalian forebrain functional plan. Each of these circuits shows specific cytoarchitectonic characteristics and are either reticularly or nuclearly organized, likely under the control of the specific expression and localization of adherence molecules (CAM, cadherins) along the corresponding pathways (for example, for the nuclearly organized amygdala and medial hypothalamic nuclei connected through the stria terminalis). Some may even be characterized by the expression of specific transcription factors (again, as illustrated for the amygdala and hypothalamus, concerning reproductive and defensive pathways—Choi et al., 2005).

“Classic” authors have already suggested that well differentiated structures of highly organized brains must have emerged during evolution from primordial reticularly organized forms. Ramon-Moliner and Nauta used of the term “phylogenetic segregation” to characterize these evolutionary processes (Ramón-Moliner and Nauta, 1966). Pre- and postoptic hypothalamic structures have been observed in amphibians, as has a dopamine rich posterior tuberculum; however, laterally organized structures cannot be found in these species. Obviously, lateral and medial hypothalamic structures are phylogenetically recent but made of neurons that derivate from phylogenetically ancient populations (see in Croizier et al., 2013 for MCH and the dorsal hypothalamus), as are the substantia nigra/ventral tegmental area in the ventral midbrain. It is functionally relevant that these structures, including the mammillary nuclei (which contain head direction cells and are part of the Papez circuit), evolved in parallel. A larger behavioral repertory in mammals, especially related to reproductive and agonistic behaviors, is related to the differentiation of the hypothalamic medial and lateral zones, but is also likely associated with increased voluntary motor controls allowed by the extrapyramidal pathways.

To conclude, it has become obvious that the classical hypothalamus with its four regions does not constitute one single neurological entity, at least from the developmental point of view. The divergence in the origins of the collection of nuclei and areas that are usually gathered between its arbitrary borders can be traced to the earliest patterns of gene expression. However, here we contend that early neurogenesis gives rise to a first mantle layer, with longitudinal and transverse components (i.e., Y-shaped) that serves as a foundation for the formation of the whole forebrain connectivity, guiding most ascending and descending tracts appearing later. These observations demonstrate that the structures of the hypothalamic region are intimately implicated within complex networks along the extrapyramidal pathway and act together for the expression of behaviors. They also suggest that these circuits that involve the telencephalon and hypothalamus share a basic organizational plan.

## Conflict of interest statement

The authors declare that the research was conducted in the absence of any commercial or financial relationships that could be construed as a potential conflict of interest.
