# Supplementary material for: Characterization of a mammalian prosencephalic functional plan
Source: Front Neuroanat. 2015 Jan 6;8:161. doi: 10.3389/fnana.2014.00161 (PMC4285092; doi:10.3389/fnana.2014.00161)
Supplement: Supplementary file 1 [file Supplementary_information.DOCX]

**Supplementary information:**

Several major tracts cross or bound the hypothalamus. They show a remarkable topographical organization (Fig. 4B). Most of these pathways can be traced from the telencephalon, and only a few extend into the caudal brainstem. From ventral to dorsal we found the following:

- The neuroendocrine ventral and periventricular pathways. Rostrally traced from GnRH producing cells in the ventral medial septal region and ventral preoptic region, this pathway also gathers projections from the anterior periventricular, paraventricular, supraoptic and arcuate nuclei and end in the neurohypophysis.

- The stria terminalis, which connects the nuclei of the amygdala (most of which are pheromonally recipient nuclei) with the medial zone nuclei of the hypothalamus and ends in the ventral premammillary nucleus.

- The hypothalamic olfactory pathway, which was very well described by Price in the 1980s (Price, 1985; Price *et al*., 1991), is also selectively labeled by anti-parvalbumin antibodies and has been characterized as the ventrolateral hypothalamic tract by some authors. Deep amygdalar nuclei project with this pathway that ends in the posterior LHA or lateral supramammillary region (nucleus Gemini of Price for olfactory projections).

- The fornix, which is generated in the dorsal subiculum and the columns of which end in the mammillary nuclei. Projections from ventral components of the hippocampal formation take a lateral and medial route through the hypothalamus, within the medial corticohypothalamic tract and medial forebrain bundle, which also contains projections from the septum.

- Finally, the ventral striatum, ventral pallidum and ventral mesencephalon are interconnected through lateral or dorsal components of the medial forebrain bundle, adjacent to the cerebral peduncle, which carries projections from the isocortex, dorsal striatum, dorsal pallidum and substantia nigra. Therefore, both the cerebral peduncle and medial forebrain bundle extend into the ventral midbrain.

Price, J.L. (1985). Beyond the primary olfactory cortex: olfactory-related areas in the cortex, thalamus and hypothalamus. *Chemical Senses*. 10, 239-258.

[Price, J.L](http://www.ncbi.nlm.nih.gov/pubmed?term=Price%20JL%5BAuthor%5D&cauthor=true&cauthor_uid=1713925)., [Slotnick, B.M](http://www.ncbi.nlm.nih.gov/pubmed?term=Slotnick%20BM%5BAuthor%5D&cauthor=true&cauthor_uid=1713925)., and [Revial, M.F](http://www.ncbi.nlm.nih.gov/pubmed?term=Revial%20MF%5BAuthor%5D&cauthor=true&cauthor_uid=1713925). (1991). Olfactory projections to the hypothalamus. [*J Comp Neurol*.](http://www.ncbi.nlm.nih.gov/pubmed/1713925) 306, 447-61.
